# Non-Gaussian Systems Control Performance Assessment Based on Rational Entropy

**DOI:** 10.3390/e20050331

**Published:** 2018-05-01

**Authors:** Jinglin Zhou, Yiqing Jia, Huixia Jiang, Shuyi Fan

**Affiliations:** 1 College of Information Science &Technology, Beijing University of Chemical Technology, Beijing 100029, China; 2 Department of Missile Engineering, Army Engineering University, Shijiazhuang 050003, China

**Keywords:** Control loop Performance Assessment, non-Gaussian system, estimation of distribution algorithm (EDA), rational entropy

## Abstract

Control loop Performance Assessment (CPA) plays an important role in system operations. Stochastic statistical CPA index, such as a minimum variance controller (MVC)-based CPA index, is one of the most widely used CPA indices. In this paper, a new minimum entropy controller (MEC)-based CPA method of linear non-Gaussian systems is proposed. In this method, probability density function (PDF) and rational entropy (RE) are respectively used to describe the characteristics and the uncertainty of random variables. To better estimate the performance benchmark, an improved EDA algorithm, which is used to estimate the system parameters and noise PDF, is given. The effectiveness of the proposed method is illustrated through case studies on an ARMAX system.

## 1. Introduction

Poor control loop performance will reduce the effectiveness of the control loop, which may lead to product quality degradation, increase product costs and other issues. There are many factors in the chemical process, which include inadequate parameter tuning and maintained controllers, equipment failure, without or insufficient feedforward compensation, inappropriate control structure design and so on, that can affect control loop performance [1]. The purpose of Control loop Performance Assessment (CPA) is to provide a comprehensive health assessment framework for control loops. Such framework includes assessing, detecting and diagnosing as well as suggesting improvement measures [2].

Harris (1989) proposed a performance indicator based on minimum variance control (MVC) [3], which laid the foundation for the development of CPA research field. Based on the MVC assessment technique, the benchmark value can be estimated from routine operational data as long as the delay is known or estimated, without additional experiments. Later researchers extended the scope of the basic Harris indicator to make the MVC-based performance assessment method suitable for feedforward/feedback control systems [4], unsteady and non-minimum phase systems [5], system for setting changes, time-varying system [6] and MIMO system [7]. Although the MVC-based controller can minimize the variance of the output, the robustness of the control system often fails to meet the requirements. In addition, constraints are often encountered during the actual production process. Therefore, Grimble et al. extended minimum variance (MV) benchmark and proposed the generalized minimum variance(GMV) algorithm to solve these problems. The GMV algorithm uses the sum of the minimized system output variance and the constrained control signal variance as an objective function and adds a dynamic minimum variance weight matrix to it [8]. Grimble (2002) used the single-step cost function to derive the derivation of the generalized minimum variance control law and proved that the error weighted amplitude should be strictly limited to ensure the stability of the system. However, the weighted selection process is not straightforward unless static weights are used.

Another extension to the MV benchmark is the linear quadratic Gaussian (LQG) benchmark [9,10,11]. Huang and Shah (1999) designed the LQG regulator as the optimal controller to replace the MVC controller to obtain the CPA index based on the LQG benchmark. It is worth noting that the main difference between the LQG benchmark and the MV benchmark is that in addition to the constraints of the system input, the LQG criterion also needs to consider both the input and the output minimum variance. Furthermore, under the same control, the LQG performance benchmark can determine how far away the current control performance is from the best performance because this performance bound can be represented by a coordination curve. However, performance assessment using the LQG benchmark is much more complex than the traditional MVC-based approach and leads to computational burden (a state estimator and the solution of algebraic Riccati equations).

In the 1970s, model predictive control (MPC) emerged in the field of process control and then many scholars introduced CPA research into it [12,13,14]. The MPC algorithm was based on a predictive model and realizes the stationary control of the controlled loop through rolling optimization and feedback correction. In [15], a different area for the CPA of Generalized predictive control (GPC) was built.

In addition, new performance assessment methods have emerged with the development of CPA research field. Such as in the literature [16,17] a Hurst index has been applied for CPA of linear feedback control loop. Compared to other performance benchmarks, it has the advantage of not requiring prior knowledge and relying only on the acquired process output data. However, different noises may have a certain influence on the calculation of the Hurst index and cause errors.

Furthermore, there are many researchers who have summarized the status of research in the area of performance assessment. Qin [18], Harris [19] and Jelali [20] had given a detailed description of CPA methods and theoretical knowledge.

Although current control performance assessment has been relatively mature in related fields. However, most of the existing CPA methods assume that noise disturbance in the system obeys a Gaussian distribution but some chemical production processes cannot satisfy this assumption. For this case, only the mean and variance as the control target in closed-loop control system can’t fully reflect the high order statistical properties. To solve these problems, minimum entropy control strategy was proposed and this control strategy has very good control effect on non-Gaussian systems [21,22,23]. There are a few research results about the minimum entropy control based CPA [24,25,26,27,28]. To avoid the shortcoming of the Shannon entropy, a rational entropy-based CPA for the output stochastic distribution control (SDC) systems was given in [24]. However, this method only gave the calculation method of the theoretical benchmark value in the SDC systems and did not give the estimation method of this benchmark. The Renyi’s entropy which is used to deal with discrete random variables was employed in References [25,26,27]. There were some theoretical deficiencies in [25] and [26]; and [27] had discretization scale selection problems. The Gaussian noise processing method was still used in [28]. Based on the above analysis, the CPA framework that directly uses the characteristic of continuous random variables for general non-Gaussian systems has not yet been established.

This paper aims to establish a benchmark minimum entropy controller to be used in the CPA of the general feedback control system with non-Gaussian disturbances. To achieve this target, an improved EDA algorithm is used to identify system parameters and estimate the distribution of disturbance and then get the minimum entropy benchmark of non-Gaussian system.

The rest of this paper is organized as follows: In Section 2, review the minimum entropy control and propose the benchmark for performance assessment. In Section 3, the performance assessment process based on EDA algorithm is introduced in detail. In Section 4, a simulation case is conducted and the conclusion is given in Section 5.

## 2. Minimum Entropy Control

### 2.1. MEC Index

**Figure 1:**
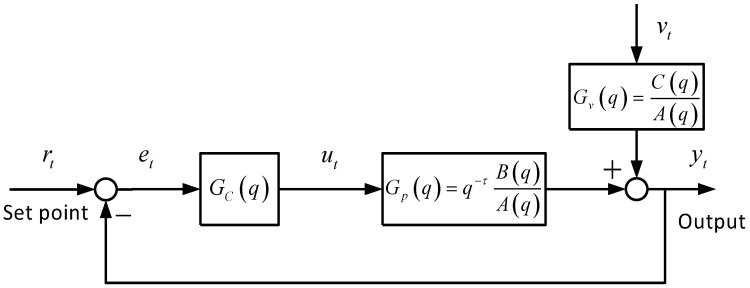
Generic feedback control system structure.

Consider a generic feedback control systems shown in Figure 1, where $r_{t}$ is the set point, $u_{t}$ is the controller output, $v_{t}$ is the unmeasured disturbance. $G_{c}$, $G_{p}$ and $G_{v}$ denote the transfer functions of the feedback controller, the process and disturbance dynamics, respectively. The set point is set to zero by convenience and the disturbances are assumed to be zero mean.

Let the system under consideration be described by an ARMAX model.

(1) $$A(q^{-1})y(t)=q^{-\tau }B(q^{-1})u(t)+C(q^{-1})v(t),$$

where y(t),u(t),v(t) are the output, the input and the noise of the ARMAX process, respectively. The disturbance transfer function $G_{v}$ in Figure 1 can be further decomposed as follows by Diophantine equation,

(2) $$G(q^{-1})=F(q^{-1})+q^{-\tau }R(q^{-1}),$$

where $F\left(q^{-1}\right)$ is the impulse response coefficients of $G_{v}$ in $q^{-1}$ with order τ−1 and $R\left(q^{-1}\right)$ is the remaining transfer function that satisfies the Identity (2).

(3) $$y(t)=Fv(t)+Lv(t-d)=\underset{\mathrm{feedback}-\mathrm{invariant}}{\underset{︸}{\left(n_{0}+n_{1}q^{-1}+n_{2}q^{-2}+\cdots +n_{d-1}q^{-(\tau -1)})v(t\right)}}+\underset{\mathrm{feedback}-\mathrm{varying}}{\underset{︸}{\left(n_{d}q^{-\tau }+n_{d+1}q^{-(\tau +1)}+\cdots )v(t\right)}},$$

where $L=\frac{R-F{\tilde{G}}_{p}G_{c}}{1+q^{-\tau }{\tilde{G}}_{pG_{c}}}$. The feedback-invariant terms are not functions of the process model or the controller; they depend only on the characteristics of the disturbance acting on the process. The second term is feedback-varying. This means that of the process output entropy (Equation (3)) depends on the structure and parameters of the controller ($G_{c}$). The entropy of the output variable can reach the minimum value if L=0.

For non-Gaussian variables, unlike Gaussian variables which have the particularity that all distribution information is contained in the first and second moments and the higher moments above the second moment are zero. So, the MVC control that only minimize the second order does not apply to the non-Gaussian systems. Fortunately, the entropy is alternative uncertainty measurement which is more general in representing the system randomness using the probability distribution that all the stochastic information is included. Therefore, all higher order moments including the second one can be optimized using the entropy instead of the mean square error optimization.

For a linear non-Gaussian system, the goal of the minimum entropy controller is to minimize the entropy of the system output variables [25,26,27,28]. Like the conventional MVC, the minimum entropy value will be obtained if and only if L=0,

(4) $$H^{\min}(y_{t})=H(Fv_{t}).$$

MEC based assessment compares the actual system-output entropy $H\left(y_{t}\right)$ to the output entropy $H^{min}\left(y_{t}\right)$ as obtained using minimum-entropy controller. And the MEC–based CPA index is represented by

(5) $$\eta =\frac{H^{\min}(y_{t})}{H(y_{t})}=\frac{H(Fv_{t})}{H(y_{t})},$$

where $H^{min}\left(y_{t}\right)$ is the entropy of the output variable with MEC and $H\left(y_{t}\right)$ is the entropy of the output variable with actual controller. This index is similar to the MVC index will be always within the interval [0,1], where MVC index values close to unity indicate good performance with regard to the theoretically achievable output minimum entropy. “0” means the worst performance, including unstable control.

In fact, the relative entropy or Kullback–Leibler (KL) divergence [29,30] can reflect the distance between two probability distributions. It is defined as follows.

$$D_{KL}(P∥Q)=\int_{-\infty }^{\infty }p(x)\log\frac{p(x)}{q(x)}dx$$

The relative entropy can be also used as a performance assessment index if p(·) denotes the probability density function (PDF) of the output variable with MEC and q(·) denotes the PDF of output variable with actual controller.

This index equal to zero indicates that the controller is the minimum entropy controller and when it deviates from zero, it is not a minimum entropy controller. However, the problem of the relative entropy-based assessment index is that the corresponding the relative entropy index is not a convex index. In other words, this index can obtain whether the current controller is the minimum entropy controller but it is difficult to find a suitable threshold to determine the current controller performance is good or bad. In this sense, it is not appropriate to use the KL distance as a control loop performance assessment index.

### 2.2. Rational Entropy

In [25,26], the authors gave a method for calculating the entropy,

(6) $$\begin{array}{c} H^{\min}(y_{t})=H(v_{t})+H(n_{1}v_{t-1})+\cdots +H(n_{\tau -1}v_{t-\tau +1})\\ =H(v_{t})+H(v_{t-1})+\cdots +H(v_{t-\tau +1}) \end{array},$$

which is based on the following lemma.

**Lemma** **1** **[25].***If*X*is a random variable, then*∀c∈R,c≠0,H(cX)=H(X).

**Lemma** **2** **[25].***For the two random variables*X*and*Y*, the entropy or the amount of information is revealed by*H[(X,Y)]=H(X|Y)+H(Y)=H(Y|X)+H(X).

If X and Y are mutually independent, H(X|Y)=H(X), then H(X,Y)=H(X)+H(Y).

However, the conclusion of Equation (6) is wrong because the two lemmas are unsuitable for the considering condition. For the Lemma 1, it is valid only for discrete random variables. But for continuous random variables, we will discuss whether Lemma 1 is established by the following examples.

Suppose the system is as follows,

(7) $$y_{t}=a_{t}+ca_{t-1}.$$

According to (6), the entropy is obtained as follows,

(8) $$\begin{array}{c} H(y_{t})=H(a_{t})+H(ca_{t-1})\\ =H(a_{t})+H(a_{t-1}) \end{array}.$$

**Figure 2:**
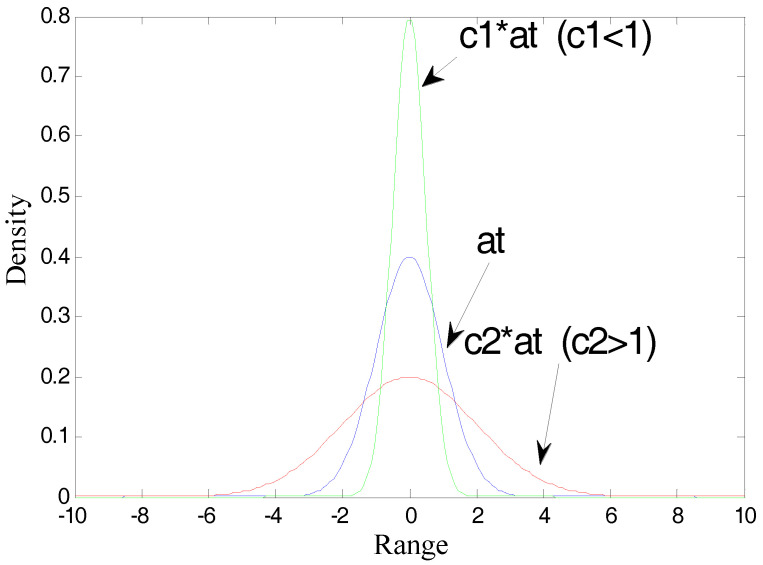
The same continuous variable distribution with different coefficients.

Then for the same distribution $a_{t}$, we use the different coefficients c to get the probability distribution as shown in Figure 2. Obviously, the different coefficients lead to the different distribution of $ca_{t}$. Since the entropy is determined by the shape of the distribution, that is, the coefficients cannot be omitted, $H\left(ca_{t}\right)\neq H\left(a_{t}\right)\left(c\neq 1\right)$.

As for the Lemma 2, probability theory shows that if the PDFs of X and Y are known and X and Y are mutually independent, the PDF of Z=X+Y is calculated as follows [28],

(9) $$f_{Z}(z)=\int_{-\infty }^{\infty }f_{X}(z-y)f_{Y}(y)dy=\int_{-\infty }^{\infty }f_{X}(x)f_{Y}(z-x)dy,$$

where $f_{X}\left(\cdot \right)$, $f_{Y}\left(\cdot \right)$ and $f_{Z}\left(\cdot \right)$ are the PDFs of X, Y and Z. The random variable Z is still a univariate variable but Lemma 2 uses the properties of multivariate random variables. The distribution of the sum of two random variables is not the same as the joint distribution. In other words, H(Z)≠H(X)+H(Y). Due to confusion of concepts, the results of the performance evaluation are not credible.

As aforementioned, the entropy is determined by the shape of the distribution (or the shape of the PDF), then the entropy of the feedback invariant is determined by the shape of the PDF of $v_{t}+n_{1}v_{t-1}+\cdots +n_{\tau -1}v_{t-\tau +1}$. In [27], a method of computing the entropy of feedback invariants called the consistent discrete distribution approximation method is introduced, which improves the deficiencies of literature [25]. Although this method ensures the unity and consistency of entropy calculation, different standards are required for different non-Gaussian noise. Therefore, the identification of distribution function is an indispensable step and it will be illustrated in subsequent sections.

In the previous studies, Shannon Entropy is one of performance assessment criteria based on minimum entropy control [28]. It is defined as,

(10) H=−∫γ(x)lnγ(x)dx,x∈R.

It seems to be a new benchmark to describe. But the Shannon entropy of the continuous random variable may be negative or even negative infinite, this means that the SE does not satisfy the “consistency” property. As a result, its uncertainty determines that it cannot be used as a new standard. Fortunately, a rational entropy (RE) instead of the SE is proposed by Zhou [24]. This type of entropy exhibits most properties of the Shannon’s entropy and, at the same time, satisfies the “consistency” property. In this paper, we use the rational entropy of the process output with MEC and the actual output to calculate performance index. Let x be a random variable in R and γ(x) be its PDF, the rational entropy (RE) is given as [24]

(11) $$H_{RE}=-\int \gamma (x)\log\frac{\gamma (x)}{1+\gamma (x)}dx.$$

Although the expression of RE is similar to that of the relative entropy, RE and relative entropy have different meanings: RE reflects the uncertainty of random variables and relative entropy reflects the distance between two probability distributions.

In fact, [24] gave a CPA index of the output stochastic distribution control (SDC) systems. However, [24] only gave the calculation method of the theoretical benchmark value and did not give the estimation method of this benchmark; On the other hand, the index of [24] was only for the SDC systems, so its theory benchmark was not generic. In other words, a CPA framework that directly uses the characteristic of continuous random variables for general non-Gaussian systems has not yet been established. This paper aims to build a MEC-based CPA index for the general feedback control system with non-Gaussian disturbances.

In the chemical process, its output is generally measurable but it is difficult to accurately obtain the process model due to the lack of complete physicochemical knowledge and random disturbance distribution. It means the actual output entropy can be obtained directly from the collected output samples by Equation (11) but the entropy of MEC process still needs to be estimated through the identification of system parameters and noise PDF estimation. To summarize, the complete algorithm to evaluate the MEC-based index and to assess feedback controls contains the steps described as follows.

(1) Select the time-series-model type. Determine/estimate the system time delay τ and system order.
(2) Identify the closed-loop model from collected output samples.
(3) Estimate the benchmark entropy of process data.
(4) Compute the performance index.

## 3. System Identification and Noise PDF Estimation

From Section 2, it is easy to know that system parameters and PDF of noise need to be accurately estimated in order to obtain RE-based CPA values.

Let $q^{-1}$ be the unit backward shift operator and define the three polynomials in $q^{-1}$ as:

(12) $$\begin{array}{l} A(q^{-1})=1+a_{1}q^{-1}+a_{2}q^{-2}+\cdots +a_{na}q^{-na}\\ B(q^{-1})=b_{1}q^{-1}+b_{2}q^{-2}+\cdots +b_{nb}q^{-nb}\\ C(q^{-1})=1+c_{1}q^{-1}+c_{2}q^{-2}+\cdots +c_{nc}q^{-nc} \end{array}.$$

Defined

(13) $$\theta =[a_{1},a_{2},\ldots ,a_{na},b_{1},b_{2},\ldots ,b_{nb},c_{1},c_{2},\ldots ,c_{nc}]$$

(14) $$h={\left[\begin{array}{ccc} -y(t-1),\ldots ,-y(t-na) & u(t-1),\ldots ,u(t-nb) & v(t-1), \end{array}\ldots ,v(t-nc)\right]}^{T}.$$

A(q), B(q) and C(q) are polynomials in $q^{-1}$ of order na, nb and nc, respectively. There are many kinds of algorithms to estimate the order [31] and delay [32] of the model. In this paper, we adopt that the order of the model is obtained by the Akaike information criterion [31]. Then, a simple and easy method, called correlation analysis, is applied here to estimate $\hat{\tau }$.

By means of indirect identification, a recursive extended least squares (RELS) algorithms is adopted then the closed loop non-Gaussian system identification is turned into open-loop Gaussian system identification. We can obtain the initial estimate of the parameters $\hat{\theta }$ and the estimation of noise variance ${\hat{\sigma }}_{v}$. Based on the estimation of the parameters of the Gaussian system, we can use the $\hat{\theta }\pm 3{\hat{\sigma }}_{v}$ as the initialized range of the parameter space. Then the improved EDA algorithm is used to obtain the system parameters and the noise distribution estimation.

The Estimation of distribution algorithm (EDA) is a population evolutionary algorithm based on statistical learning theory. The probability model is used to describe the distribution information of candidate solutions in the search space. A statistical learning method is used to establish a probabilistic model describing the distribution from the perspective of population. Then, a random sampling of the estimated probability distribution model is used to generate some new individuals to replace some of the individuals with poor fitness values in the initial population to form a new generation population. When satisfying the iteration termination condition, the iteration of this algorithm will be terminated and finally the optimal outcomes obtained by employing EDA are the best fitness value of the current population.

Based on the traditional EDA algorithm, the parameters of preliminary estimation and data selection are added to improve the speed of searching and optimization precision. And the system parameter identification problem is transformed into the optimization problem in high dimensional parameter space. In order to make the parameter space covers the real parameters as much as possible, the preliminary estimation is used to determine the range of initialization parameter space.

Then, we adopt $\hat{\theta }$ as seeds. In order to eliminate the seeds with large deviations, adding the screening conditions. Since the mean of the noise is assumed to be zero, the mean of the residuals can be regarded as the screening condition. It can be described as follows.

(15) $$\begin{array}{c} e_{t}=y_{t}-h^{T}\theta \\ mean(e_{t})<\varepsilon \;(\varepsilon >0) \end{array}.$$

Finally, we take the error entropy as the fitness value, when the error entropy reaches the minimum while the parameter reaches the optimum. The improved EDA algorithm is summarized as follows.

**Figure 3:**
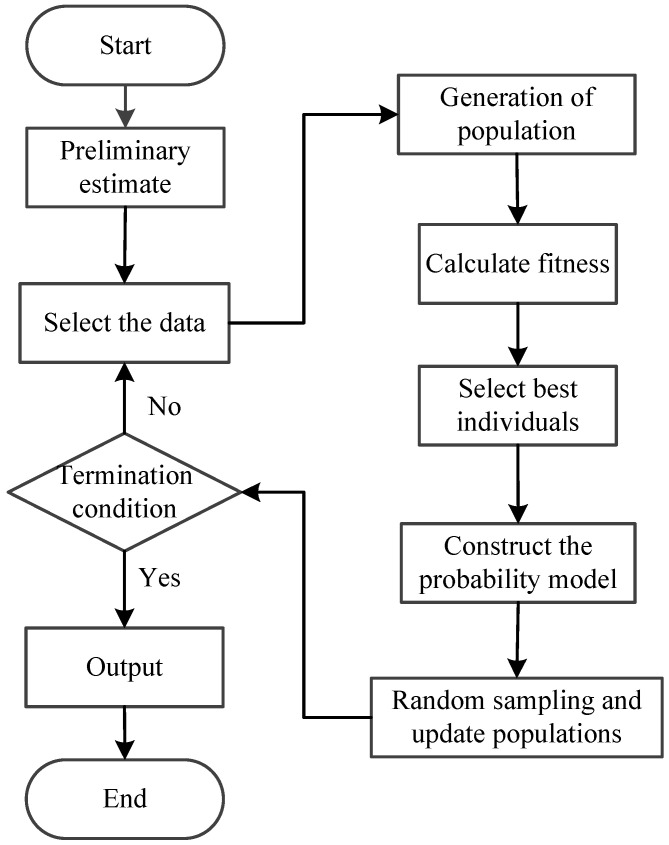
The process of the improved distribution estimation algorithm.

(1) Preliminary estimation. Rough estimation parameters are obtained by recursive maximum likelihood method. Then the task of initializing the parameters space can be accomplished with a uniform distribution using the initial value of parameter as the value range.
(2) Screen seeds. From the first generation randomly select R group seed from the parameter space and calculate the mean value of the residuals. Remove the seeds whose mean value is more than ε and keep the seeds whose mean value is less than ε. If the number of reserved seeds is less than R, re-sampling is performed until the number of seeds retained is not less than R and then the seeds reserved are collected in new parameter spaces $\Psi^{\left(l\right)}\left(Q\right)\left(Q\;\geq \;R\right)$.
(2) Calculate fitness. Randomly create R individuals from the parameter space $\Psi^{\left(l\right)}\left(Q\right)\;A_{l}=\left\{\Phi^{\left(l\right)}\left(1\right),\Phi^{\left(l\right)}\left(2\right),\ldots ,\Phi^{\left(l\right)}\left(R\right)\right\}$. The error entropy of the parameter vector in $A_{l}$ is estimated based on the selected training data set $\left\{y_{i}\right\}$.
(4) Select N superior individuals $B_{l}=\left\{\Phi^{\left(l\right)}\left(1\right),\Phi^{\left(l\right)}\left(2\right),\ldots ,\Phi^{\left(l\right)}\left(N\right)\right\}$ based on the cost function and estimate PDF based on the error entropy extracted from the selected N individuals.
(5) Calculate the estimated average value of the selected N parameters and establish the probability models.
(6) Set l←l+1 and resample R individuals from the updated PDF.
(7) Go to step 2 until the stopping criterion is met. This process can be represented by Figure 3.

Through the above process, not only the distribution of noise ${\hat{v}}_{t}$ can be estimated but also the coefficient $\hat{F}$ of feedback invariant can be obtained by calculating the Diophantine equation according to the result of parameter identification. And then we can estimate the relationship between output and disturbance under ME control. That is, ${y_{t}}^{mec}=\hat{F}{\hat{v}}_{t}$. Meanwhile, the MEC-based CPA index can be estimated as follows,

(16) $$\hat{\eta }=\frac{H(y_{t}^{mec})}{H(y_{t})}=\frac{H^{\min}(\hat{F}{\hat{v}}_{t})}{H(y_{t})}.$$

## 4. Simulation

In order to illustrate the effectiveness of the proposed method, the following system which is the same as that of [25,27] is considered.

$$y\left(t\right)=u\left(t-2\right)+\frac{1-0.2z^{-1}}{1-z^{-1}}v\left(t\right).$$

The transfer function of the controller is chosen as $G_{c}=\frac{K}{1-0.2z^{-1}-0.8z^{-2}}$. From this given system, it is easy to know that the system parameters are θ=[−1, 1, −1, −0.2] and τ=2. The parameters of feedback-invariant terms is F=[1, 0.8] which can be obtained by solving the Diophantine equation with the nominal parameters.

In all simulation cases, the parameters of improved EDA algorithm is set as N=80, R=200. And a Gaussian model with diagonal covariance matrix is utilized as the probabilistic model. The selection criteria is ${\bar{e}}_{t}<=0.1$. The stopping criteria is set to $\left|H^{\left(l\right)}-H^{\left(l-1\right)}\right|<0.001$ where $H^{\left(l\right)}=H\left({\bar{e}}^{\left(l\right)}\right)$ denotes the error entropy obtained at lth based on the estimated average parameters.

To demonstrate more clearly, the section is divided as follows: In Section 4.1, parameters estimation and CPA with a fixed controller and unimodal distribution noises is discussed. Then keep all parameters unchanged, a simulation experiment is carried out for the bimodal distribution noises in Section 4.2 and the proposed CPA indices with different gain controllers are given in Section 4.3.

### 4.1. Parameters and CPA Index Estimation with Fixed Controller and Unimodal Distribution Noises

In this simulation, the gain of the controller is chosen as K=1.2. $v_{t}$ is assumed to follow a unimodal distribution including normal distribution N(0,0.255), Beta distribution B(2,9) and exponential distribution E(0.5). The corresponding parameter estimation results are as follows

(1) Normal distribution
  $$\hat{\theta }=\left[-0.9219,\;0.9247,\;-0.9239,\;-0.0733\right]$$
  $$\hat{F}=\left[1,\;0.8486\right]$$
(2) Beta distribution
  $$\hat{\theta }=\left[-0.9732,\;0.9921,\;-0.9930,\;-0.0910\right]$$
  $$\hat{F}=\left[1,\;0.8822\right]$$
(3) Exponential distribution
  $$\hat{\theta }=\left[-0.9190,\;0.9432,\;-0.9422,\;-0.1105\right]$$
  $$\hat{F}=\left[1,\;0.8085\right]$$

The results show that the parameters of the ARMAX model can be estimated by the improved EDA algorithm under different noises. However, we are more concerned with the noise estimated values and its distribution.

**Figure 4:**
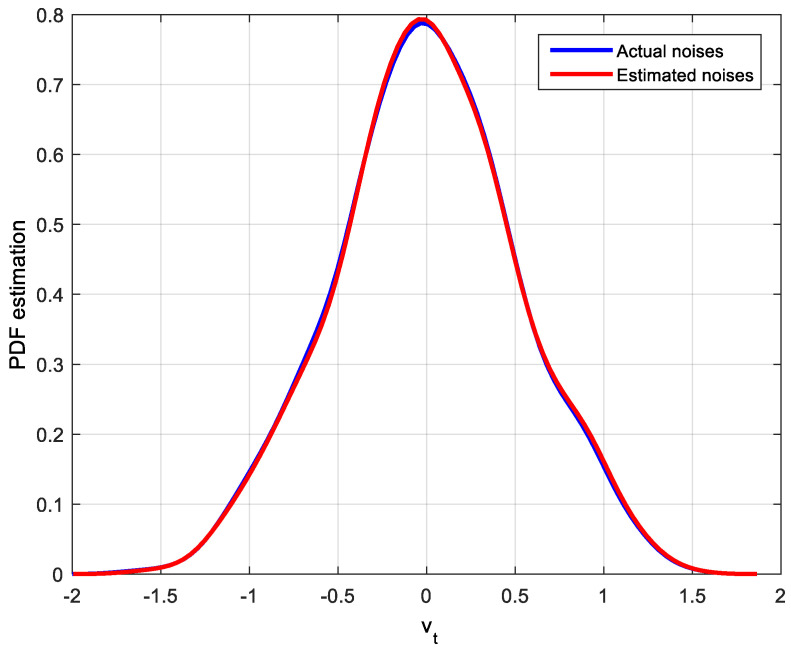
PDF estimation of the actual and estimated Gaussian distribution noises.

**Figure 5:**
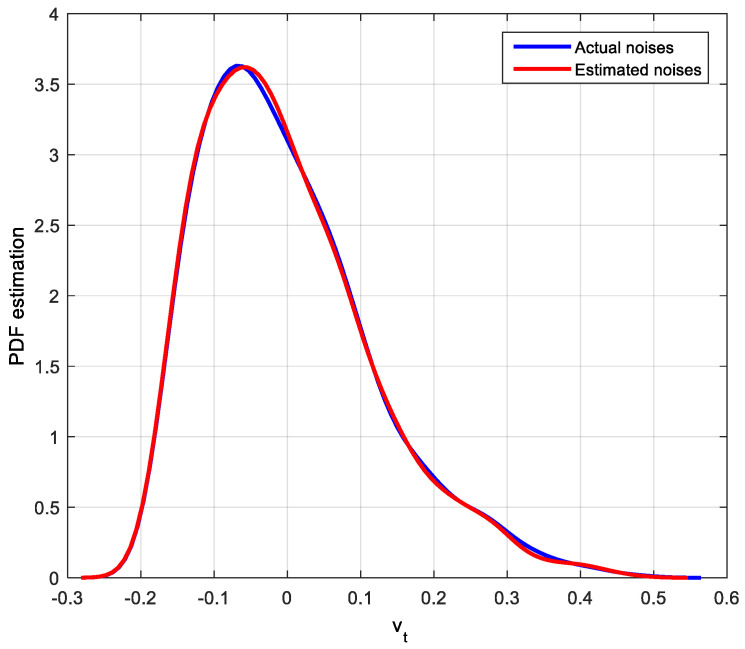
PDF estimation of the actual and estimated Beta distribution noises.

**Figure 6:**
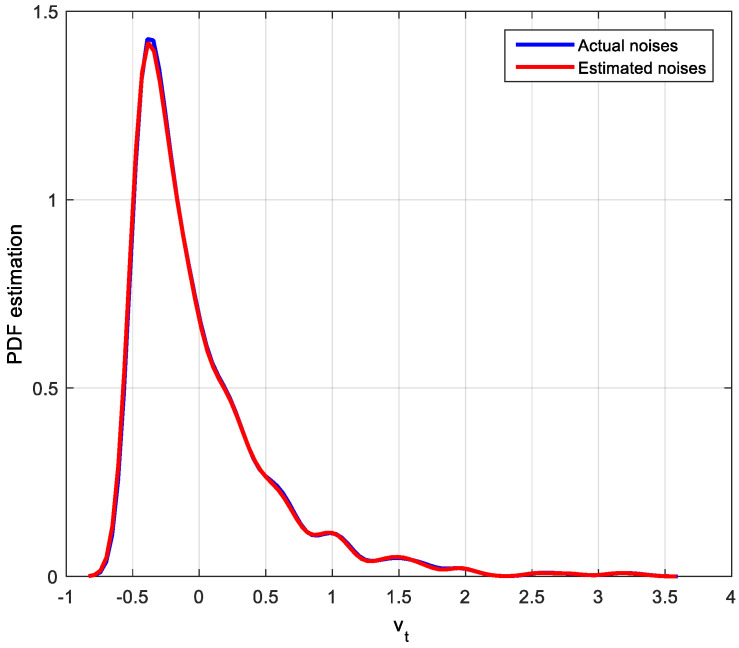
PDF estimation of the actual and estimated Exponential distribution noises.

Noise estimated values can be obtained by the improved EDA algorithm. There are many ways to describe the estimation effect, such as histogram and kernel density estimation. Since PDF is used in the MEC-based CPA index, the kernel density estimation method for actual noises and estimated noises will be used to show the estimation effect. The corresponding PDF estimation results of actual noises and estimated noises are shown in Figure 4, Figure 5 and Figure 6.

After identification by the improved EDA algorithm, not only the parameters of the system are obtained but also the distribution of noise is estimated. It is clear that the estimated disturbance distribution is close to the true system disturbance distribution. Hence, the simulation results in this case demonstrate the efficiency of the improved EDA algorithm. With this distribution and the feedback-invariant estimation, the MEC-based indices can be easily computed as shown in Table 1.

Based on Table 1, the CPA index under MVC and MEC maintain at around 0.82 and 0.94, respectively. It indicates that when the system noise obeys the Gaussian distribution or the other unimodal distribution, there is no significant difference between the minimum variance and the minimum entropy criterion.

To illustrate that MEC-based CPA index is more applicable than MVC-based under the circumstances where the variance may fail. Bimodal distribution noises are selected as an example in the next subsection.

### 4.2. Parameters and CPA Index Estimation with Fixed Controller and Bimodal Distribution Noises

Keep all parameters except the distribution of $v_{t}$ unchanged and the distribution of $v_{t}$ is chosen as the following bimodal distribution.

$$v\sim f\left(x\right)=a\cdot \frac{r}{\sigma_{1}\sqrt{2\pi }}e^{-\frac{{\left(x-\mu_{1}\right)}^{2}}{2\sigma_{1}^{2}}}+b\cdot \frac{1-r}{\sigma_{2}\sqrt{2\pi }}e^{-\frac{{\left(x-\mu_{2}\right)}^{2}}{2\sigma_{2}^{2}}}$$

where $\mu_{1}=-3,\mu_{2}=3,\;\sigma_{1}=1,\;\sigma_{2}=0.4.\;r=0.2,\;0.4\;\mathrm{or}\;0.6$. To make comparisons, the RELS identification algorithm is also applied here. And the parameter estimation results are as follows.

(1) r=0.2
  $${\hat{\theta }}_{EDA}=\left[-1.0210,1.0231,-1.0229,-0.1922\right]$$
  $${\hat{F}}_{EDA}=\left[1,\;0.8289\right]$$
  $${\hat{\theta }}_{RELS}=[-0.9785,\;1.0562,-1.0429,-0.2213$$
(2) r=0.4
  $${\hat{\theta }}_{EDA}=\left[-0.9150,\;0.9317,-0.9318,-0.1104\right]$$
  $${\hat{F}}_{EDA}=\left[1,\;0.8045\right]$$
  $${\hat{\theta }}_{RELS}=\left[-0.9650,\;0.9523,\;-0.9500,\;-0.2039\right]$$
(3) r=0.6
  $${\hat{\theta }}_{EDA}=\left[-0.9142,\;0.9475,-0.9471,-0.1136\right]$$
  $${\hat{F}}_{EDA}=\left[1,\;0.8006\right]$$
  $${\hat{\theta }}_{RELS}=\left[-0.9211,\;0.9221,-0.9209,-0.1694\right]$$

**Figure 7:**
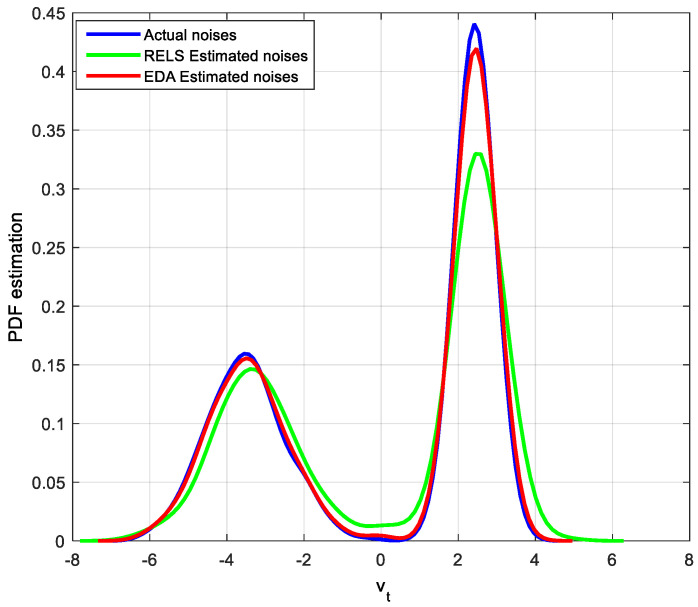
PDF estimation of the actual and estimated bimodal distribution noises.

Parameter estimation results show that both methods seem to be available as system parameter estimates. However, the noise and its estimated distribution shown in Figure 7 indicate that the RELS method is ineffective in estimating the noise of the bimodal distribution.

Figure 7 shows that that the proposed method is also effective for the bimodal non-Gaussian system identification. According to the parameter identification results, the CPA value can be calculated as shown in Table 2.

Comparing the data in Table 1 and Table 2, the estimated system performance using the entropy index is consistent where the noise obeys above distributions. Simulation results show that the performance assessment method based on minimum entropy can effectively evaluate noise obeying random distribution system and reduce the limitations of the existing evaluation methods.

Although in the framework of different noise and the same controller, the proposed MEC-based CPA index can give a consistent conclusion, is there similar consistency for different controllers? We will discuss this problem next.

### 4.3. CPA Index with Different Gain Controllers

In this simulation, the controller gain K will be changed to get the controller performance trends. K=0.8 is the ME controller. In this subsection, the method of CPA index estimation in [27] is used here for comparison. And two scales (0.05 and 0.01) [27] are selected to the CPA index estimation as shown in Table 3 and Table 4, respectively, and the corresponding CPA index estimation value with the proposed method is given in Table 5. Moreover, the CPA index estimation value with the proposed method for other controllers is given in Table 6.

Table 3, Table 4 and Table 5 provide performance assessment results of the different controllers. When the system controller selects the optimal controller, the system output entropy can get to the minimum value, which means that the system output $y_{t}$ is equivalent to the system’s feedback invariant and the corresponding CPA index is very close to 1. Meanwhile, with the increase of K, the CPA values estimated by the two methods the have the same monotonous decreasing trend whether they are unimodal distribution or bimodal distribution. But it is noticeable in Table 3 that there are certain CPA values (the blue ones) which give wrong evaluation conclusions. This is because this large discretization scale (0.05) is not suitable for this type of bimodal distribution. Furthermore, it is easy to see from Table 5 and Table 6 that the proposed CPA index can well reflect the performance of the current controller regardless of whether the control gain increases or the control gain decreases. The above simulation results show that the proposed scheme is more suitable for non-Gaussian systems CPA.

## 5. Conclusions

In this paper, by analyzing the limitation of MVC-based CPA method, a new MEC-based CPA method is developed for linear non-Gaussian system and the entropy index is obtained by PDF estimation. Furthermore, the improved EDA algorithm is adopted to estimate the parameters and noise distribution of the system and then calculate the entropy of the feedback invariant. The effectiveness of the CPA procedures is tested by many simulations. It can be concluded that the new MEC-based CPA index can be used to assess the control loop performance of linear systems with non-Gaussian stochastic disturbance. However, the efficiency of the improved EDA algorithm is still an open problem. Further research will focus on optimization algorithms or consider other strategies for parameter identification and PDF estimation.

## Figures and Tables

**Table 1:** CPA index values using MVC and MEC benchmark.

	N(0,0.255)	B(2,9)	E(0.5)
$\eta_{\mathrm{MV}}$	0.8396	0.8256	0.8282
${\hat{\eta }}_{\mathrm{MV}}$	0.8416	0.8251	0.8282
$\eta_{\mathrm{ME}}$	0.9457	0.9375	0.9400
${\hat{\eta }}_{\mathrm{ME}}$	0.9464	0.9452	0.9417

**Table 2:** CPA index values under bimodal distribution disturbance.

	r=0.2	r=0.4	r=0.6
$\eta_{\mathrm{ME}}$	0.9447	0.9201	0.9323
${\hat{\eta }}_{\mathrm{ME}}$	0.9359	0.9176	0.9294

**Table 3:** CPA index values with the different gain controller [27].

		K=0.8	K=1.0	K=1.2	K=1.4	K=1.6
N(0,0.255)	η	1.0000	0.9903	0.9611	0.9096	0.8186
	$\hat{\eta }$	1.0060	0.9965	0.9669	0.9151	0.8238
B(2,9)	η	0.9999	0.9829	0.9369	0.8625	0.7418
	$\hat{\eta }$	1.0094	0.9932	0.9464	0.8718	0.7487
bimodal r=0.2	η	1.0001	0.8971	0.8188	0.7725	0.7615
	$\hat{\eta }$	1.0540	0.9336	0.8539	0.8033	0.8044
bimodal r=0.4	η	0.9992	0.9142	0.8616	0.8479	1.0216
	$\hat{\eta }$	1.0389	0.9430	0.8948	0.8853	1.0688
bimodal r=0.6	η	0.9999	0.9487	0.9106	0.9161	1.0901
	$\hat{\eta }$	1.0170	0.9674	0.9210	0.9302	1.1060

**Table 4:** CPA index values with the different gain controller [27].

		K=0.8	K=1.0	K=1.2	K=1.4	K=1.6
N(0,0.255)	η	1.0000	0.9979	0.9924	0.9829	0.9667
	$\hat{\eta }$	0.9953	0.9933	0.9878	0.9783	0.9622
B(2,9)	η	1.0000	0.9967	0.9883	0.9747	0.9353
	$\hat{\eta }$	0.9960	0.9927	0.9844	0.9708	0.9496
bimodal r=0.2	η	1.0000	0.9746	0.9482	0.9271	0.9064
	$\hat{\eta }$	1.0010	0.9761	0.9509	0.9302	0.9098
bimodal r=0.4	η	1.0000	0.9776	0.9592	0.9457	0.9309
	$\hat{\eta }$	1.0009	0.9800	0.9611	0.9475	0.9330
bimodal r=0.6	η	1.0000	0.9855	0.9712	0.9606	0.9481
	$\hat{\eta }$	0.9998	0.9854	0.9713	0.9612	0.9488

**Table 5:** CPA index values with different gain controller using the proposed method.

		K=0.8	K=1.0	K=1.2	K=1.4	K=1.6
N(0,0.255)	η	0.9999	0.9607	0.9477	0.8286	0.7169
	$\hat{\eta }$	0.9981	0.9429	0.9421	0.8178	0.7124
B(2,9)	η	0.9919	0.9512	0.9310	0.8242	0.7102
	$\hat{\eta }$	0.9871	0.9405	0.9212	0.8095	0.7260
bimodal r=0.2	η	0.9944	0.9833	0.9350	0.8821	0.7210
	$\hat{\eta }$	0.9925	0.9741	0.9350	0.8767	0.7158
bimodal r=0.4	η	0.9978	0.9618	0.9209	0.8626	0.7481
	$\hat{\eta }$	0.9982	0.9589	0.9194	0.8644	0.7495
bimodal r=0.6	η	0.9989	0.9819	0.9207	0.8996	0.7299
	$\hat{\eta }$	0.9987	0.9798	0.9212	0.8994	0.7289

**Table 6:** CPA index values with different gain controller using the proposed method.

		K=0.7	K=0.6	K=0.5	K=0.4
N(0,0.255)	η	0.9389	0.9394	0.9281	0.9000
	$\hat{\eta }$	0.9477	0.9208	0.9169	0.9186
B(2,9)	η	0.9428	0.9391	0.9026	0.8055
	$\hat{\eta }$	0.9357	0.9250	0.8901	0.8021
bimodal r=0.2	η	0.9816	0.9591	0.9423	0.9042
	$\hat{\eta }$	0.9802	0.9560	0.9562	0.9117
